# Minimising the impact of stable ^208^Pb on recovery of ^212^Pb from a generator

**DOI:** 10.1186/s41181-025-00357-4

**Published:** 2025-07-30

**Authors:** Rachel Roberts, Tim Carthy, Jennifer Young, Temi Awogboro, Howard Greenwood, Jane Sosabowski, Scott Heath, Francis Livens

**Affiliations:** 1 Department of Chemistry, The University of Manchester, Oxford Rd, Manchester, M13 9PL UK; 2 United Kingdom National Nuclear Laboratory, Springfields, Salwick, Preston, Lancashire PR4 0XJ UK; 3 Centre for Cancer Biomarkers and Biotherapeutics, Barts Cancer Institute, Queen Mary University of London, London, EC1M 6BQ UK; 4 School of Biomedical Engineering and Imaging Sciences, King’s College London, London, SE1 7EH UK; 5 Department of Earth and Environmental Sciences, The University of Manchester, Oxford Rd, Manchester, M13 9PL UK

**Keywords:** Lead, Specific activity, Molar activity, Radiolabelling, Stable lead

## Abstract

**Background:**

Stable ^208^Pb will accumulate over time in all ^212^Pb generators, forming an increasing proportion of the Pb atoms and potentially impacting the efficiency of chelation of ^212^Pb. This paper models the accumulation of ^208^Pb in ^212^Pb-parent generators with time, calculates the optimal elution time with respect to effective specific activity (ESA) of the ^212^Pb produced, and considers whether the presence of ^208^Pb could prevent the required molar activities (A_m_) being achieved. A matrix calculation was utilised to model the decay of ^224^Ra and ^228^Th parents in generators and the associated accumulation of ^208^Pb with time. This model was used to calculate the optimal elution times for each generator with respect to ESA and A_m_.

**Results:**

The optimal elution time with respect to A_m_ of ^212^Pb is 16.5 h for a ^224^Ra generator (61.0% of ^224^Ra starting activity, A_m_ = 6997 MBq ^212^Pb/nmol total Pb) and 19.2 h for a ^228^Th generator (71.4% of ^228^Th starting activity, A_m_ = 6566 MBq ^212^Pb/nmol total Pb).

**Conclusions:**

The limited data available in literature shows the level of ^208^Pb present in these studies does not prevent attainment of the required molar activities for pre-clinical or clinical work. However, with other chelators and under other conditions this may not be the case. The authors would encourage others to measure and report the level of ^208^Pb in ^212^Pb generator eluates alongside radiolabelling results and the max A_m_ of the radiopharmaceutical ^212^Pb (vector molecule (VM)) achieved.

**Supplementary Information:**

The online version contains supplementary material available at 10.1186/s41181-025-00357-4.

## Introduction

**Fig. 1:**
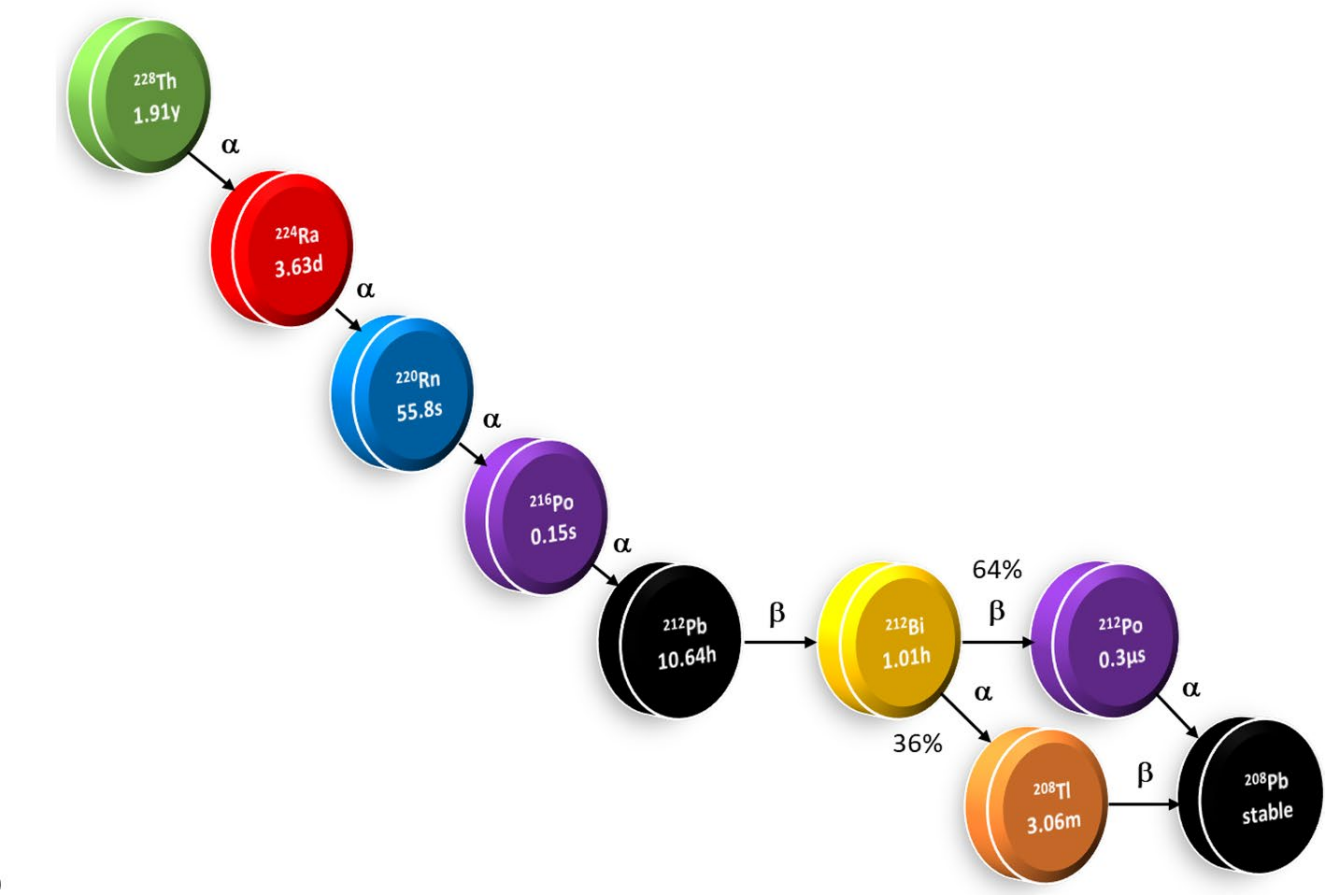
Decay chain of ^228^Th down to stable ^208^Pb, half-life data taken from JEFF 3.3 database

The demand for ^212^Pb for use in Targeted Alpha Therapy (TAT) is expected to increase substantially over the next 5–10 years and many production routes are under development globally to meet future demand (Zimmermann 2024). Increasing availability of the ^212^Pb-generating parent radionuclides, ^224^Ra and ^228^Th, is driving innovation in generator design with ^212^Pb isolated either by removal from an exchange substrate using a suitable eluent (Atcher et al. 1987; Mirzadeh 1998; Pruszynski et al. 2021), or by capture of ^220^Rn emanation from the parent radionuclide and recovery of the decay product ^212^Pb (Hassfjell 2001; Li et al. 2023; Boldyrev et al. 2018). However, neither method is capable of separating ^212^Pb from its stable daughter ^208^Pb (Fig. 1), which will accumulate on or in the chosen substrate and, over time, will form an increasing proportion of the Pb atoms present as ^212^Pb (t_1/2_ = 10.64 h) grows in to reach the activities required for pre-clinical studies or clinical use. This reduces the measured amount of radioactivity per mole of Pb, which in turn has an impact on the final radiopharmaceutical product, which will contain a mixture of ^212^Pb-vector molecule (^212^Pb-VM) and ^208^Pb-vector molecule (^208^Pb-VM).

The decay of parent radionuclide, either ^228^Th or ^224^Ra, within a ^212^Pb generator occurs through a chain of daughter radionuclides with each parent atom eventually resulting in an atom of stable ^208^Pb. Whilst alpha emissions within the chain decrease mass number, the total atom count across the chain is conserved. As such, the accumulation of ^208^Pb atoms can be calculated through an atom count of all the intermediate radionuclides present.

For simple radioactive decay a solution of the classic Bateman equations (Bateman 1910) can be used:

1 $$N_{n} \left( t \right) = N_{1} \left( 0 \right) \times \left( {\mathop \prod \limits_{i = 1}^{n - 1} \lambda_{i} } \right) \times \mathop \sum \limits_{i = 1}^{n} \frac{{e^{{ - \lambda_{i} t}} }}{{\mathop \prod \nolimits_{{\begin{array}{*{20}c} {j = 1,} \\ {j \ne i} \\ \end{array} }}^{n} \left( {\lambda_{j} - \lambda_{i} } \right)}}$$

Equation 1 denotes a solution of the Bateman equations. Where t is time, n, i & j are nuclide indices (with n = 1 being the parent nuclide), and λ is the nuclide decay constant. Equation 1 is a solution for a single parent nuclide present at time t = 0 with no branching in the decay chain. Where several radionuclides are decaying together, beginning out of secular equilibrium and/or where chain branching is involved, these equations are more complex and are typically programmed into a computational code. The fuel depletion code FISPIN is employed within the nuclear industry to calculate the composition of irradiated fuel and associated waste streams. FISPIN is a complex tool which requires specific user training, has limited availability, and is far more sophisticated than required in this case. As such, a method has been developed in this work, based on the solution to the classic Bateman equations described in Ladshaw et al. (Ladshaw et al. 2020), which is shown to align with a FISPIN run code, with the aim of providing a more accessible tool to show the composition of lead isotopes within different ^212^Pb generators with time.

The ratio of radioactive ^212^Pb and stable ^208^Pb present within a solution at a point in time can be expressed as either the effective specific activity (ESA of ^212^Pb) or molar activity (A_m_ of ^212^Pb) as defined below in Eqs. 2 and 3 with units of Bq/g and Bq/mol respectively:

2 $$ESA{}_{{}}^{212} Pb = \frac{{Activity \left( {{}_{{}}^{212} Pb} \right)}}{{mass \left( {{}_{{}}^{212} Pb} \right) + mass \left( {{}_{{}}^{208} Pb} \right)}}$$

Equation 2 denotes the definition of effective specific activity for ^212^Pb used.

3 $$A_{m} {}_{{}}^{212} Pb = \frac{{Activity{}_{{}}^{212} Pb}}{{Moles \left( {{}_{{}}^{212} Pb} \right) + Moles \left( {{}_{{}}^{208} Pb} \right)}}$$

Equation 3 denotes the definition of molar activity for ^212^Pb used.

The value of the ESA or the A_m_ of ^212^Pb at the point that the solution is used for radiolabelling, is a factor which could impact on the radiolabelling results achieved. Whilst ^212^Pb generators are in the development stage it is valuable to understand the factors in both generator design and their use that contribute to the ESA and A_m_ of the solution these generators produce, including the ^208^Pb derived from the decay of ^212^Pb—the focus of this paper.

The aim of developing ^212^Pb generators is to provide a convenient local source of ^212^Pb solution with appropriate properties for radiolabelling ^212^Pb-radiopharmaceuticals. The ideal properties of generators have been reviewed in Radiation Technology Series and No. 5 (2025). Briefly, these generators should be designed to allow efficient separation of the daughter radionuclide from the parent radionuclide; separation should be fast and meet the purity specification; elution volume/radioactivity concentration should be suitable for radiolabelling procedure; and ideally the radioactive eluate should be able to be used directly in the radiolabelling reaction without further processing/buffer exchange.

When conducting and optimising radiolabelling, conditions should be chosen to maximise the amount of ^212^Pb-radiopharmaceutical (^212^Pb(VM)) present in the final solution, and minimise, as much as reasonably practicable, the three other components (a) unbound ^212^Pb, (b) unlabelled radiopharmaceutical-precursor or vector molecule (VM), or (c) the vector molecule to a non-radioactive metal (i.e. ^208^PbVM). Unbound ^212^Pb should be limited to prevent accumulation in non-target cells, causing off-target radiotoxicity (Milenic et al. 2015). For this reason, radiopharmaceuticals typically have strict quality control specifications that limit the level of unbound radiometal present in the final formulation. The vector molecule (VM) and the vector molecule bound to a non-radioactive metal (^208^Pb(VM)) must also be limited as it could block the receptors of interest and therefore reduce the uptake of ^212^Pb(VM) at desired sites, in turn reducing the effectiveness of the therapeutic radiopharmaceutical (Luurtsema et al. 2021; Velikyan 2014). Other factors that drive the minimisation of precursor are; (a) cost considerations, as this is often an expensive component to manufacture and (b) there is a risk that the precursor could start to have undesired pharmacological properties if administered above a certain threshold level, although this would be dependent on the precursor in question (Luurtsema et al. 2021; Velikyan et al. 2008).

The ratios of these four components can be expressed by stating both the radiochemical purity and the molar activity of the solution at a specific point in time. These are defined for a radiopharmaceutical in Eq. 4 below with units of %:

4 $$Radiochemical\;purity = \frac{{Activity\; {}_{{}}^{212} Pb\left( {VM} \right)}}{{Activity\;{}_{{}}^{212} Pb\left( {VM} \right) + Activity\;{}_{{}}^{212} Pb}}$$

Equation 4 denotes definition of radiochemical purity used.

Radiochemical purity will need to be kept as high as possible for a therapeutic pharmaceutical (circa > 97%) to control off-target effects. This requirement is non-negotiable—and impacts on the molar activity achievable at this defined radiochemical purity, with molar activity defined in Eq. 5 below with units of MBq/mole:

5 $$Molar\;activity {}_{{}}^{212} Pb\left( {VM} \right) = \frac{{Activity\; {}_{{}}^{212} Pb\left( {VM} \right)}}{{Moles\; {}_{{}}^{212} Pb\left( {VM} \right) + Moles\;{}_{{}}^{208} Pb\left( {VM} \right) + Moles \;VM}}$$

Equation 5 denotes the definition of molar activity for ^212^Pb(VM) used.

As can be seen from the molar activity equation increasing the level of ^208^Pb in the solution will reduce the A_m_ through the production of ^208^Pb(VM). As ^212^Pb and ^208^Pb are chemically identical the VM will bind to them in the same manner and they will compete with each other, so as the level of ^208^Pb in the solution increases the total amount of VM required to achieve the same radiochemical purity specification increases, as there must be enough to bind to all of the ^208^Pb as well as the ^212^Pb.The achievable A_m_ of ^212^PbVM at a defined activity level and radiochemical purity will be influenced not only by the level of ^212^Pb and ^208^Pb but by other factors including: (1) *The activity concentration of the radiolabelling solution* (MBq/mL); (2) *The concentration of the precursor present* (nmol/mL); (3) *The number of chelator molecules attached to each precursor and the chelator affinity for the radiometal*. Generally, for small molecules and peptides there is just one chelator per precursor, but for antibodies there can be multiple (Baidoo et al. 2013). The chelator affinity for the radiometal impacts how much excess chelator is required to bind all the ^212^Pb in solution. In a model system each chelator would have one ^212^Pb atom bound– but in the conditions required for radiolabelling, an excess of chelator drives equilibrium towards complexation due to the very low concentrations of metal ions in radiolabelling solutions (Tsionou et al. 2017; Holland 2018). The higher the affinity of the chelator for the metal, the lower the level of excess required to bind all of the ^212^Pb present. This can also be referred to as the chelator occupancy—the percentage of the chelator molecules present which have a ^212^Pb attached; Typical chelator occupancy levels can range from 0.01 to 10% depending on the conditions (Müller et al. 2023; McNeil et al. 2021, 2023; Lee et al. 2024; Westrøm et al. 2017) (4) *The presence of competing metals*. Competing metals will block available chelators and stop the maximum occupancy being achieved with ^212^Pb. The more specific the chelator is for Pb the less other metals will influence radiolabelling outcomes, however stable ^208^Pb will always compete with ^212^Pb as it is chemically identical. For this reason, the A_m_ of ^212^Pb is an important factor to be considered when radiolabelling, and insights into how this can be increased, such as through selecting the best elution time, or oversizing a ^212^Pb generator by using higher parent activity than ^212^Pb dose activity required, as described in this paper could be invaluable when optimising radiolabelling conditions to maximise the A_m_ of ^212^Pb(VM).

In order to provide context for impact that the levels of ^208^Pb present in eluant from ^212^Pb generators may have when radiolabelling with ^212^Pb, it was useful to estimate molar activities which would likely be needed to be achieved when working with ^212^Pb radiopharmaceuticals. The values for clinical production selected were based on the max activities used in the phase 1 ^212^Pb trial which used ^212^Pb-DOTAM-TATE (250 MBq per patient (Delpassand et al. 2022)) and due to limited information in this paper about the radiochemistry procedure, supplemented with values for the production of ^177^Lu-DOTA-TATE (Advanced Accelerator Applications 2018), due to the two tracers presumed similarities (150 nmol). The values for the preclinical (5 MBq, 0.5 nmol) were selected from a number of literature sources (Rold et al. 2020; Saidi et al. 2024).

From this it was estimated that > 10 MBq/nmol would be needed for a preclinical study and > 3 MBq/nmol for a clinical study, and so these values are used as indicative benchmarks within this paper.

## Methods

### Matrix method

For any given decay chain, the nuclides can be sorted in order of decreasing mass number (ensuring that parent nuclides always have lower indices than their progeny) and indexed accordingly. Table 1 illustrates this for the ^232^U chain as an example.

**Table 1:** Nuclides of the ^232^U decay chain using data from (Tuli 2011)

Index	Nuclide	Half-life (years)	λ (s^−1^)
0	^232^U	6.89 × 10^01^	3.19 × 10^−10^
1	^228^Th	1.91 × 10^00^	1.15 × 10^−08^
2	^224^Ra	9.94 × 10^−03^	2.21 × 10^−06^
3	^220^Rn	1.76 × 10^−06^	1.25 × 10^−02^
4	^216^Po	4.59 × 10^−09^	4.79 × 10^00^
5	^212^Pb	1.21 × 10^−03^	1.81 × 10^−05^
6	^212^Bi	1.15 × 10^−04^	1.91 × 10^−04^
7	^212^Po	9.44 × 10^−15^	2.33 × 10^06^
8	^208^Tl	5.82 × 10^−06^	3.77 × 10^−03^
9	^208^Pb	1.00 × 10^100^	2.20 × 10^−108^

Note that although the referenced paper (Ladshaw et al. 2020) describes a separate matrix solution for stable nuclides, for mathematical simplicity in this work, any stable nuclides in a chain (e.g. ^208^Pb) are given arbitrarily long (but unique within the chain) half-lives. This avoids singularities in the population functions (Eqs. 7 and 8) and generates decay constants for the stable nuclides that are so small as to be insignificant in terms of subsequent calculations on applicable timescales.

Once so arranged and given a vector for the starting conditions, the atom count, *z*, for any nuclide *i* at any time *t* can be obtained using Eq. 6 taken from p8. of Ladshaw et al. (2020):

6 $$z_{i}^{t} = \mathop \sum \limits_{j \le i}^{{}} \left( {\mathop \sum \limits_{k = j}^{i} v_{i}^{k} u_{k}^{i} e^{{ - \lambda_{k} t}} } \right)z_{j}^{0}$$

Equation 6 denotes the atom count z of nuclide i at time t. Where *t* is time in seconds, *i*,* j* and *k* are nuclide indices, λ_k_ is the decay constant for nuclide *k*, z^0^ is the vector of initial atom counts for each nuclide at *t* = 0.

*v* & *u* are lower triangular matrices whose values are generated using the population functions described below in Eqs. 7 and 8 respectively from p. 7. of Ladshaw et al. (2020).

7 $${\text{v}}_{{\text{i}}}^{{\text{k}}} = \left\{ {\begin{array}{*{20}l} 0 \hfill & {{\text{for}}\;{\text{i}} < {\text{k}}} \hfill \\ 1 \hfill & {{\text{for}}\;{\text{i}} = {\text{k}}} \hfill \\ { - \frac{1}{{\uplambda _{{\text{k}}} -\uplambda _{{\text{i}}} }}\mathop \sum \limits_{{{\text{j}} < {\text{i}}}} {\text{v}}_{{\text{j}}}^{{\text{k}}} {\text{b}}_{{{\text{ij}}}}\uplambda _{{\text{j}}} } \hfill & {{\text{for}}\;{\text{i}} > {\text{k}}} \hfill \\ \end{array} } \right.$$

Equation 7 denotes the population function for matrix *v*.

where *b* is the branch fraction matrix, and b_*i,j*_ is the branch fraction of the *j*th nuclide that forms the *i*th nuclide.

8 $${\text{u}}_{{\text{i}}}^{{\text{k}}} = \left\{ {\begin{array}{*{20}l} 0 \hfill & {{\text{for}}\;{\text{i}} < {\text{k}}} \hfill \\ 1 \hfill & {{\text{for}}\;{\text{i}} = {\text{k}}} \hfill \\ { - \mathop \sum \limits_{{{\text{j}} < {\text{i}}}} {\text{u}}_{{\text{j}}}^{{\text{k}}} {\text{v}}_{{\text{i}}}^{{\text{j}}} } \hfill & {{\text{for}}\;{\text{i}} > {\text{k}}} \hfill \\ \end{array} } \right.$$

Equation 8 denotes the population function for matrix *u*.

The derivation of Eqs. 6–8 is beyond the scope of this work. Details of the matrix algebra involved can be found in Ladshaw et al. (2020, pp. 4–8).

**Fig. 2:**
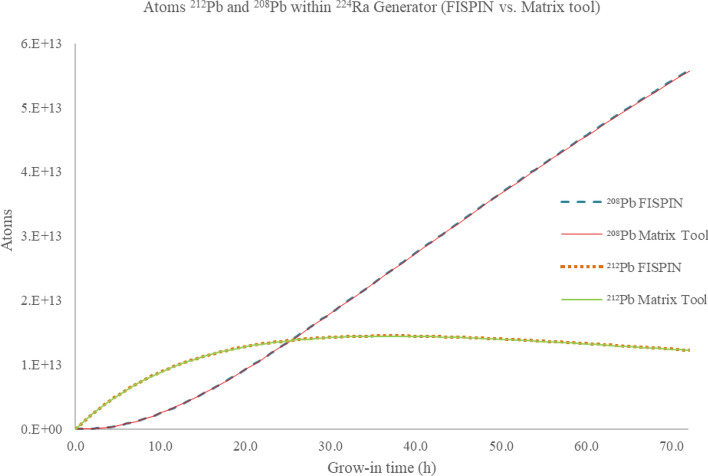
Atoms of ^208^Pb and ^212^Pb growing into a ^224^Ra generator (assumed 350 MBq starting activity of ^224^Ra) against time (h) using both FISPIN and Matrix tools

These functions were implemented in Mathcad Prime 8 and the atom count results obtained validated by checking against those from manual calculations and outputs from FISPIN (see Fig. 2).

It is a relatively simple matter to export the constant value matrices v & u generated in Mathcad Prime for a given decay chain to an Excel workbook, define a suitable variable for z^0^ and implement Eq. 6 in Visual Basic for Applications (VBA). The atom count of stable nuclide(s) can be confirmed by subtracting total atoms of unstable radionuclides present within the chain at specified time intervals, from the starting atoms of parent radionuclide. A work through of the method for the ^232^U chain is provided in Supplementary Material 1.

The results presented below were obtained using the Matrix method above, for the following scenarios where “grow-in” time refers to the time elapsed since the previous ^212^Pb elution:

- 350 MBq ^224^Ra generator having undergone a ^212^Pb (and daughter) elution at 100% efficiency, at grow-in times between 0 and 50 h,
- 350 MBq ^228^Th generator having undergone a ^212^Pb (and daughter) elution at 100% efficiency, at grow-in times between 0 and 50 h,
- ‘Oversized’ ^228^Th generators (550, 600, 700, 800, 900 and 1000 MBq) having undergone a ^212^Pb (and daughter) elution at 100% efficiency, on grow-in of 500 MBq ^212^Pb activity,
- the decay of a 350 MBq ^212^Pb eluate post- elution at various allowed grow-in periods (10, 12, 24 and 48 h), assuming 100% elution efficiency.

The modelled scenarios, using the matrix tool, assume the elution at time zero removes all ^212^Pb along with all ^212^Bi, ^212^Po, ^208^Tl and ^208^Pb daughters and do not account for the presence of other Pb isotopes which could result from the production method of parent radionuclide. The generator sizes in scenarios (a), (b) were selected based on a ^224^Ra generator loaded with 370 MBq (available from the United States Department for Energy Isotope program), allowing for some decay during transport (National Isotope Development Center 2025).

### Optimal elution times

The grow-in time required to recover the maximum possible activity of ^212^Pb from ^224^Ra and ^228^Th based generators was estimated using Eq. 9 (Le et al. 2014) where λ_1_ and λ_2_ are the decay constants for parent and daughter radionuclides respectively:

9 $$t_{max} = \frac{{\left[ {\ln \left( {\lambda_{2} /\lambda_{1} } \right)} \right]}}{{\left( {\lambda_{2} - \lambda_{1} } \right)}}$$

Equation 9 denotes the time of maximum grow-in activity for daughter radionuclide to parent generator.

According to Le et al. the optimal elution time of a parent generator can be calculated with respect to grow-in time t_opt(t)_ or effective specific activity t_opt(ESA)_ by finding the maximum of the mean progress functions described in Le et al. (2014) and given below in Eqs. 10 and 11:

10 $$f \left( {A,t} \right) = \frac{A}{{\left( {t/A} \right)}}$$

Equation 10 denotes the mean progress function for optimisation of daughter nuclide grow-in versus grow-in time

11 $$f\left( {A,ESA} \right) = \frac{A}{{\left( {1/ESA} \right)}} = \frac{A}{{\left( {N/A} \right)}}$$

Equation 11 denotes the mean progress function for optimisation of daughter nuclide grow-in versus effective specific activity. Where (A) activity of daughter radionuclide, (t) grow-in time, (ESA) effective specific activity of daughter radionuclide, (N) accumulating atoms of daughter radionuclide and stable daughter. The maxima of these functions give the respective optimal elution times.

## Results

### Matrix tool comparison with FISPIN

To validate the matrix method an identical calculation was performed using both the matrix method and FISPIN 10.2.1 (FISGUI version 1.1, using JEF 2.2 nuclear data libraries) for the decay of a 350 MBq ^224^Ra generator over a 72-h period. The ingrowth of ^212^Pb and ^208^Pb atoms from both calculations are plotted against time since previous Pb elution in Fig. 2, where the two outputs are in clear agreement. During the first 25 h of grow-in atoms of ^212^Pb exceed ^208^Pb, with atom ratio ^212^Pb:^208^Pb of 1:0.981 (FISPIN) and 1:0.984 (Matrix tool) at 25 h, after which ^208^Pb increasingly comes to dominate the isotope ratio giving a ^212^Pb:^208^Pb atom ratio of 1:4.532 (FISPIN) and 1:4.551 (Matrix tool) at 72 h grow-in.

### Elution of modelled scenarios

**Fig. 3:**
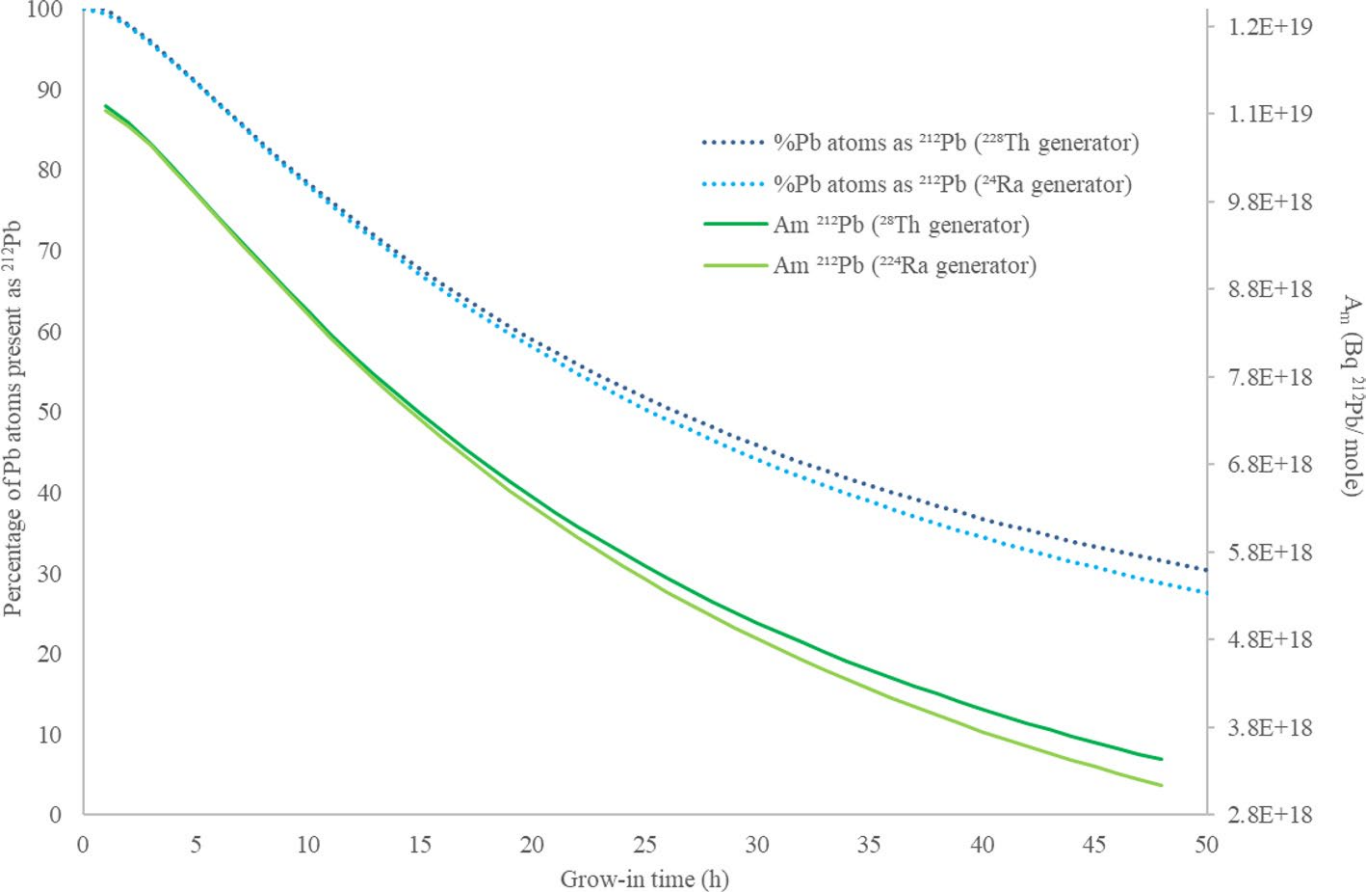
Percentage of Pb atoms present as ^212^Pb on ^224^Ra (light blue) and ^228^Th (dark blue) generators, and molar activity (A_m_) with time (h) from previous elution, calculated using the matrix tool. Independent of generator size

The proportion of Pb atoms on a generator present as ^212^Pb, as a function of time since previous Pb elution, was calculated using the matrix tool, given in Fig. 3 for both ^224^Ra- and ^228^Th-based generators, independent of generator size, along with molar activity (in terms of ^212^Pb activity (Bq) per mole total Pb).

At 24 h grow-in for a ^224^Ra generator, 51.8% of the Pb atoms present are ^212^Pb, but at 48 h grow-in this is reduced to 28.8%. There is a very slight advantage in the case of a ^228^Th (t_1/2_ = 1.01 y) generator, with 53.1% as ^212^Pb at 24 h and 31.5% as ^212^Pb at 48 h grow-in from previous elution. This slight difference is due to the activity of ^212^Pb being sustained with the longer parent half-life of ^228^Th whereas decay of parent activity in a ^224^Ra generator begins to impact on the recoverable activities of ^212^Pb.

This effect does not have a significant impact on the A_m_ of ^212^Pb removed from a ^224^Ra (light green) or ^228^Th (dark green) generator. Despite the higher activities which can be eluted from a ^228^Th generator, the accumulation of ^208^Pb atoms over time is proportionately higher too, such that the ESA of ^212^Pb (per g of total Pb) is only slightly higher in ^228^Th generator eluates than those of a ^224^Ra generator.

**Fig. 4:**
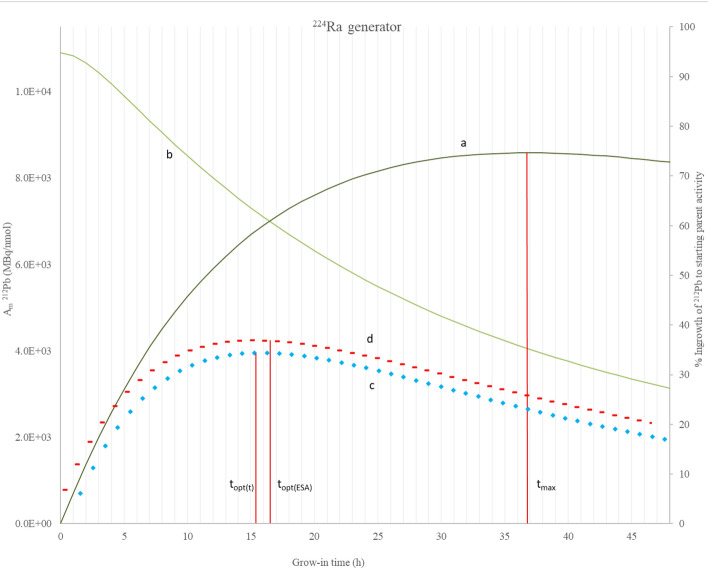
**a** Percentage ingrown activity relative to starting ^224^Ra activity on secondary axis, **b** Molar activity of ^212^Pb eluate (MBq ^212^Pb/nmol Pb), **c** mean progress function of the ^212^Pb grow-in versus time f(A,t), **c** mean progress function of the ^212^Pb grow-in versus effective specific activity f(A,ESA), **d** plotted against grow-in time (h)

**Fig. 5:**
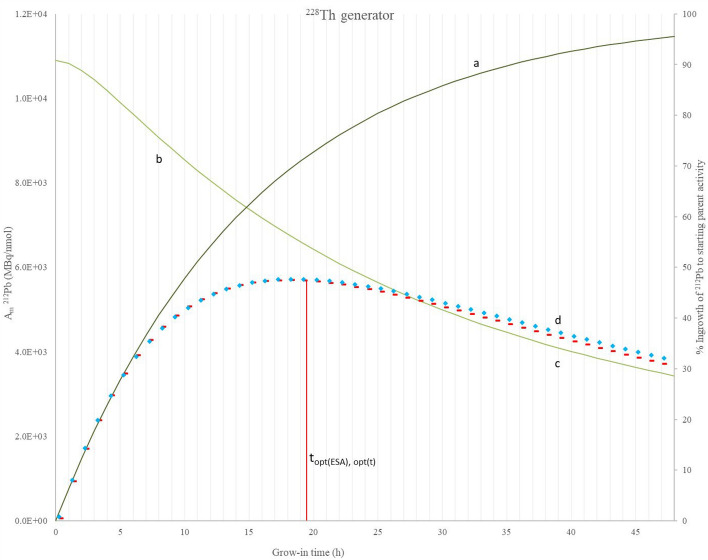
**a** Percentage ingrown activity relative to starting ^228^Th activity on secondary axis, **b** Molar activity of ^212^Pb eluate (MBq ^212^Pb/nmol Pb), **c** mean progress function of the ^212^Pb grow-in versus time *f*(A,t), **d** mean progress function of the ^212^Pb grow-in versus effective specific activity *f*(A,ESA), all plotted against grow-in time (h)

The percentage ingrowth of ^212^Pb to parent activity (a—secondary axis), A_m_ of ^212^Pb (b—primary axis), mean progress function of ^212^Pb grow-in versus time (c—x axis only) and mean progress function of ^212^Pb grow-in versus effective specific activity (d—x axis only) are plotted against grow-in time in Figs. 4 and 5 for ^224^Ra and ^228^Th generators respectively. These calculations are generator size independent.

To optimise the ^212^Pb effective specific activity it is advantageous to remove ^212^Pb from its parent radionuclide before establishing secular equilibrium. The grow-in time required to recover the maximum possible activity, as a single batch of ^212^Pb (t_max_), was calculated as 36.7 h (74.6% of ^224^Ra starting activity, ESA = 1.95 × 10^16^ Bq/g, A_m_ = 4.066 MBq/nmol) for a ^224^Ra generator and 113.1 h (99.8% of ^228^Th starting activity, ESA = 7.18 × 10^15^ Bq/g, A_m_ = 1501 MBq/nmol) for a ^228^Th generator using Eq. 9 (Le et al. 2014).

The optimal elution time with respect to grow-in time (t_opt(t)_) was calculated as 15.4 h (58.9% of ^224^Ra starting activity, ESA of 3.42 × 10^16^ Bq/g, A_m_ = 7211 MBq/nmol) for a ^224^Ra generator, and 19.2 h (71.4% of ^228^Th starting activity, ESA of 3.12 × 10^16^ Bq/g, A_m_ = 6566 MBq/nmol) for a ^228^Th generator. The optimal elution time with respect to effective specific activity (t_opt(ESA)_), inclusive of ^208^Pb atoms, was calculated as 16.5 h (61.0% of ^224^Ra starting activity, ESA = 3.32 × 10^16^ Bq/g, A_m_ = 6997 MBq/nmol) for a ^224^Ra generator, and 19.2 h for a ^228^Th generator. Note that for a ^228^Th generator t_opt(t)_ = t_opt(ESA)_ which is in accordance with the conclusion from Le et al. (2014), that where the ratio of decay constants λ_2_/λ_1_ > 1000, the ratio t_opt(t)_/t_opt(ESA)_ becomes 1.

**Fig. 6:**
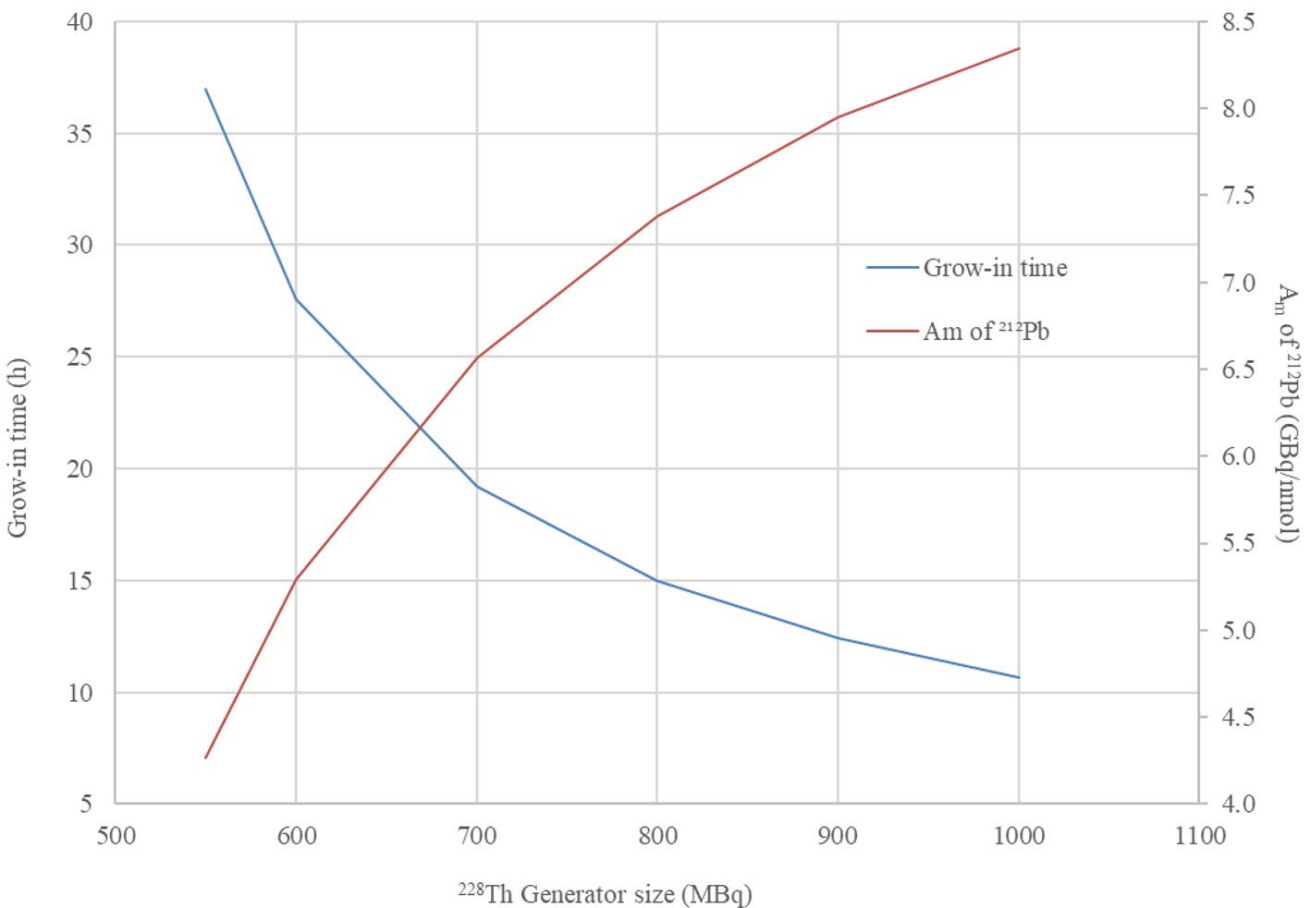
Grow-in time required for the elution of 500 MBq ^212^Pb (primary axis), and A_m_ (GBq/nmol) of eluted ^212^Pb (secondary axis), from various sized ^228^Th generators

Whilst the t_opt(ESA)_ for each generator calculated by this method is the optimal elution time from the perspective of most efficient use of parent activity, a considerably higher A_m_ and ESA of ^212^Pb can be achieved with shorter grow-in times with use of higher parent activity. Where a fixed activity of ^212^Pb is required, the A_m_ and ESA could be improved by using generators oversized in parent activity with respect to the activities of daughter required, given that earlier elution limits the accumulation of stable ^208^Pb. The required grow-in time and A_m_ of ^212^Pb eluates for various sized ^228^Th based generators eluted at grow-in of 500 MBq ^212^Pb are given within Fig. 6. In this case oversizing a ^228^Th generator to 1 GBq would allow elution of the required activity of ^212^Pb at 10.7 h (A_m_ = 8.35 × 10^18^, ESA = 3.97 × 10^16^ Bq/g), whereas a 550 MBq ^228^Th generator will require 37.0 h grow-in to reach 500 MBq ^212^Pb at a lower molar activity (A_m_ = 4.27 × 10^18^, ESA = 2.04 × 10^16^ Bq/g).

**Fig. 7:**
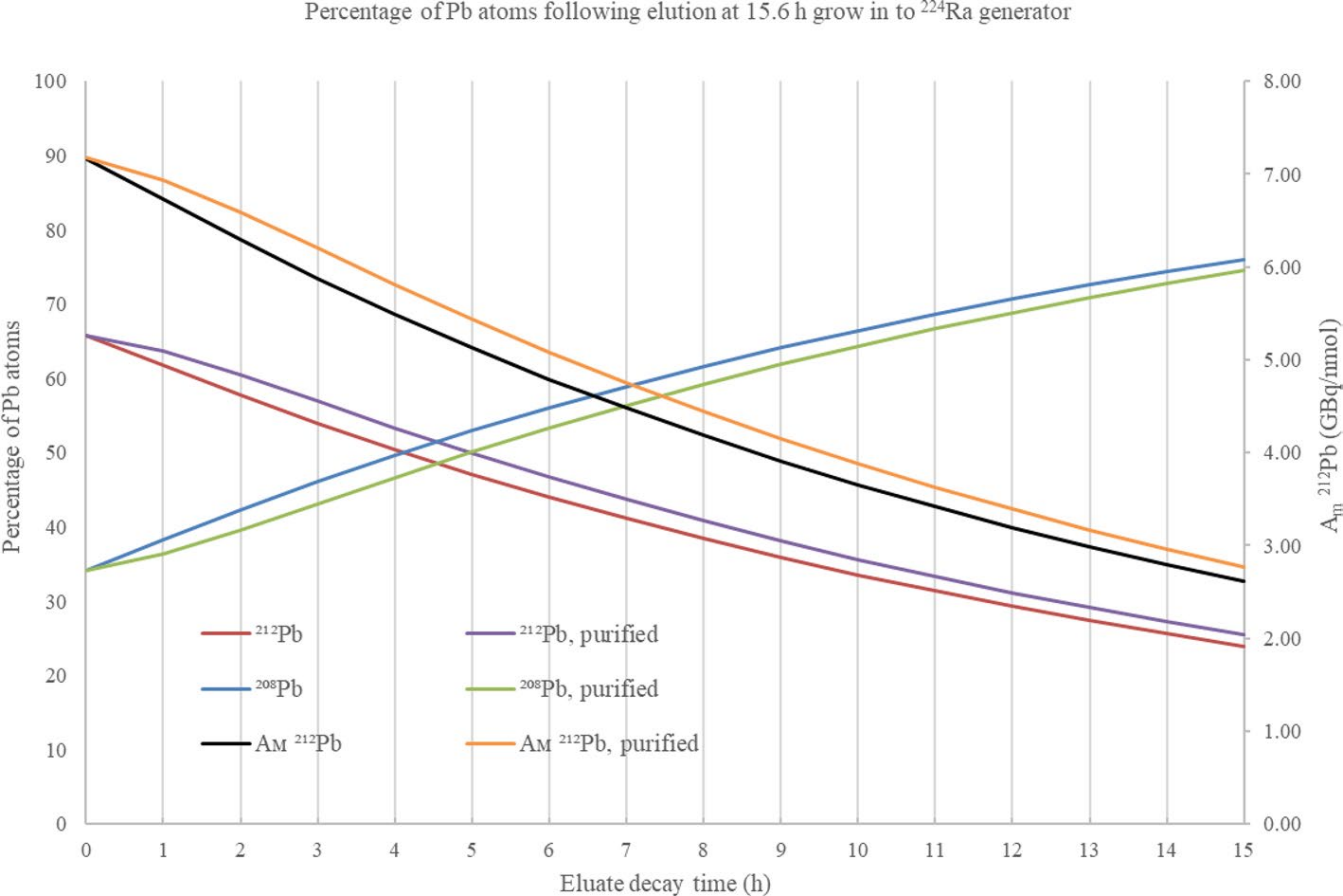
Proportion of Pb atoms present as ^212^Pb and ^208^Pb where daughters are included (^212^Pb-red, ^208^Pb-blue) and excluded (^212^Pb-purple, ^208^Pb-green), with time (h) following elution from a ^224^Ra (size independent) generator after a 15.6 h grow-in period of ^212^Pb all plotted on the primary axis. A_m_ of ^212^Pb eluate where daughters are included (black) and excluded (orange) plotted on the secondary axis

**Fig. 8:**
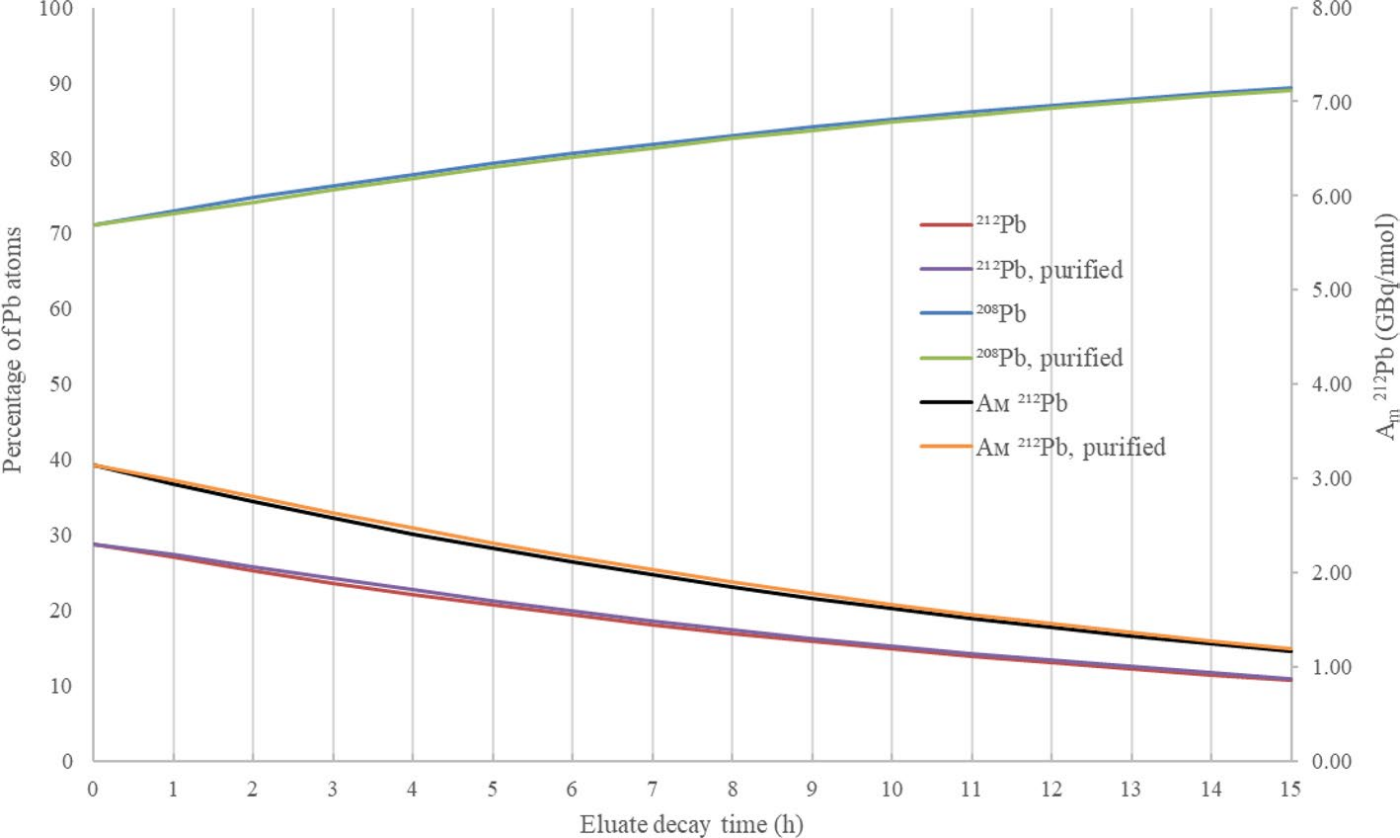
Proportion of Pb atoms present as ^212^Pb and ^208^Pb where daughters are included (^212^Pb-red, ^208^Pb-blue) and excluded (^212^Pb-purple, ^208^Pb-green), with time (h) following elution from a ^224^Ra (size independent) generator after a 48.0 h grow-in period of ^212^Pb all plotted on the primary axis. A_m_ of ^212^Pb eluate where daughters are included (black) and excluded (orange) plotted on the secondary axis

It is also important to consider the accumulation of ^208^Pb resulting from the decay of ^212^Pb following elution from a generator. The primary axis of Figs. 7 and 8 show the percentage of Pb atoms present as ^212^Pb and ^208^Pb within eluates removed from a ^224^Ra generator after grow- in times from previous elution of 415.6 h (Fig. 7, calculated optimal elution time) and 48 h (for comparison). These percentages are applied within Figs. 7 and 8 to compare the A_m_ of eluates removed, plotted on the secondary axis against decay time following elution. A pessimistic assumption was applied during these calculations, where all ^212^Pb daughters are eluted and remain with the ^212^Pb product. Figures 7 and 8 provide a comparison of scenarios where daughters are removed immediately following elution, and as such cannot contribute to the accumulation of ^208^Pb. In the case of an eluate removed after 15.6 h grow-in there is a slight improvement in the A_m_ of ^212^Pb which is calculated at 5 h decay as 5.44 GBq/nmol where daughters are removed by purification, and 5.13 GBq/nmol without removal. For eluates collected after longer grow-in periods as in Fig. 8 this effect is minimised due to the ^208^Pb contribution from daughter atoms being overwhelmed by that accumulated and removed from the generator.

The location of a ^212^Pb generator in relation to the site of radiolabelling must be considered in estimating the extent of eluate decay and ^208^Pb grow in following elution. For a generator located within a hospital based radiopharmacy, it is reasonable to consider a 1–2 h processing time following elution for quality assurance and drug formulation. A generator held within a centralised radiopharmacy, requiring transport of the ^212^Pb to a site for administration could be subject to several hours further decay. A ^212^Pb eluate removed from a ^224^Ra generator at 15.6 h grow-in, without purification, will start with an atom ratio of 1:0.519 (^212^Pb:^208^Pb, A_m_ = 7.17 GBq/nmol) and, assuming a 5-h decay time to the site of radiochelation, this ratio changes to 1:1.124 (^212^Pb:^208^Pb, A_m_ = 5.13 GBq/nmol). In comparison, an eluate removed at 48 h grow-in will decay from 1:2.473 (^212^Pb:^208^Pb, A_m_ = 3.14 GBq/nmol) to 1:3.833 (^212^Pb:^208^Pb, A_m_ = 2.41 GBq/nmol) over the same 5-h period.

## Discussion

### ***Optimised elution of ***^***212***^***Pb generators***

There is no distinct advantage observed in the ESA or A_m_ of ^212^Pb eluates, with respect to ^208^Pb content, obtained from ^228^Th generators over equally sized ^224^Ra generators when eluted at the respective optimal elution times t_opt(ESA)_. However, where using generators which are over-sized in relation to the required ^212^Pb dose, and eluting prior to the t_opt(ESA)_ for higher actual A_m_ (as in Fig. 6), there is a practical benefit in use of the longer-lived, sustained parent activity of ^228^Th, as opposed to oversizing a ^224^Ra-based generator which, within days would no longer be ‘oversized’. This advantage may go some way to balancing the practical and safety considerations associated with safe containment of ^228^Th and its progeny in siting a generator. All scenarios modelled within this paper assume complete elution (100%) of ^212^Pb which is considered slightly optimistic in the operation of most ^212^Pb generators. Most calculations also assume the complete elution of daughter radionuclides ^212^Bi, ^208^Tl and ^212^Po, which may be overly pessimistic given that with suitable purification methods the daughters could be eliminated. The effect of this assumption is shown in Fig. 7 to have a more significant effect on the estimated A_m_ of ^212^Pb eluted at earlier grow-in times.

The assessments carried out above consider ^212^Pb eluted from ^224^Ra and ^228^Th based ion exchange type generators. There are a number of ^212^Pb generator designs emerging which operate through capture of the gaseous ^228^Th and ^224^Ra decay daughter, ^220^Rn (t_1/2_ = 55.8 s). These generators collect ^220^Rn through constant removal and accumulate decay daughters settled on a substrate to be washed off, or capture ^220^Rn directly into solution (Hassfjell 2001; Li et al. 2023). Consequently, these generators require at least the same accumulation time as column-based generators, and when considering that capture of ^220^Rn is not completely efficient within ^220^Rn based generators (Hassfjell 2001), the longer time required for accumulation of required activities of ^212^Pb will lead to a higher proportion of ^208^Pb and lower A_m_ of ^212^Pb.

### Impact on radiolabelling

Currently, there is no reported data on the impact of ^208^Pb levels on radiolabelling with ^212^Pb over a range of isotope ratios, but experiments have demonstrated that for radiolabelling with ^203^Pb the presence of ^208^Pb impacts the molar activity achievable (McNeil et al. 2023). In the published data on ^203^Pb, a reduction in the ^208^Pb present from 2.4 to 0.2 nmol/mL gave an increase in molar activity for the radiopharmaceutical [^203^Pb][Pb(Crypt-OH)]^2+^ from 0.75 to 7 MBq/nmol (McNeil et al. 2023).

Through the modelling of ^212^Pb generators reported here, it was calculated that the molar activity of the ^212^Pb ranges from a theoretical maximum of 10,898 MBq/nmol (100% ^212^Pb 0% ^208^Pb) at t = 0, to 3005 MBq/nmol (28% ^212^Pb, 72% ^208^Pb) at t = 50 h. This is a 3.5-fold difference in A_m_ of ^212^Pb and it therefore follows, if all of the other conditions were maintained in order to hit the same specifications, that 3.5 fold more precursor would be required to radiolabel the same activity of ^212^Pb with this lower molar activity, reducing the max A_m_ of ^212^Pb(VM) achievable by this same ratio.

The impact of the presence of this level of ^208^Pb on ^212^Pb-radiolabelling can be assessed theoretically assuming a 10%, 1%, 0.1% or a 0.01% chelator occupancy, and compared to the indicative values we estimated will be required for pre-clinical and clinical work (10 MBq/nmol and 3.3 MBq/nmol). These calculated values are shown in Table 2. As discussed above the chelator occupancy achievable is driven by the chelator affinity for the radiometal, plus the other experimental conditions. The results in Table 2 show that, when the percentage occupancy is around 0.1%, the level of ^208^Pb present has an important impact on the ability to hit the required A_m_ of ^212^Pb(VM) values and so is a critical factor that needs to be controlled. If the occupancy is at 1% or 10% the A_m_ of ^212^Pb(VM) will meet the specifications defined at either level of ^208^Pb, and at 0.01% occupancy even an A_m_ of ^212^Pb(VM) of 10,898 MBq/nmol, equivalent to 100% ^212^Pb, would not be sufficient to meet the specifications.

**Table 2:** The calculated molar activity (A_m_ of ^212^Pb(VM)) at different occupancies at the molar activities of ^212^Pb eluate (A_m_ of ^212^Pb) achieved at t = 0 h, t = 50 h and the literature value measured experimentally (McNeil et al. 2021)

	A_m_ of ^212^Pb MBq/nmol	Ratio of ^212^Pb to ^208^Pb	Calculated A_m_ of ^212^Pb(VM) at 10% occupancy	Calculated A_m_ of ^212^Pb(VM) at 1% occupancy	Calculated A_m_ of ^212^Pb(VM) at 0.1% occupancy	Calculated A_m_ of ^212^Pb(VM) at 0.01% occupancy
	MBq/nmol	%	MBq/nmol	MBq/nmol	MBq/nmol	MBq/nmol
Modelling prediction t = 0	10,898	100% ^212^Pb, 0% ^208^Pb	**1089.8**	**109.0**	**10.9**	*1.1*
Modelling prediction t = 50 h	3108	28% ^212^Pb, 72% ^208^Pb	**310.8**	**31.1**	*3.1*	*0.3*
Literature values	1000	9% ^212^Pb, 91% ^208^Pb	**100**	**10**	*1*	*0.1*

Bold values indicate where the specification of 10 MBq/nmol and 3.3 MBq/nmol are met and italic values indicate when these specifications are not met

The level of ^208^Pb present in ^212^Pb radiolabelling solutions has been reported in one paper at 4 ppb which is equivalent to 0.02 nmol/mL (McNeil et al. 2023). This solution had been eluted after 2 days of ^212^Pb build-up on the generator, and at the time of radiolabelling had an activity concentration of 20 MBq/mL. The molar activity A_m_ of ^212^Pb was therefore 1000 MBq/nmol (9% ^212^Pb, 91% ^208^Pb). This A_m_ of ^212^Pb is three times lower than our calculations indicated would be present after the build-up of ^208^Pb on a generator for 50 h (3108 MBq/nmol 28% ^212^Pb, 72% ^208^Pb) and suggests that the ^208^Pb present in the solution tested experimentally may have come from sources other than the decay chain of ^212^Pb.

Possible sources of additional ^208^Pb include ion exchange resins (inside the generator or used for purification), buffers or solvents used during radiolabelling and laboratory equipment. A particular cross-contamination risk in radiochemistry labs is the use of lead shielding for radiation protection. It has been reported that ^208^Pb is also one of the key stable metal impurities in ^68^Ga-generator eluates at concentrations of 0.1–1 nmol/mL (Cusnir et al. 2019; Velikyan et al. 2004) and this was postulated to be from lead shielding. Care should therefore be taken to ensure that any radiochemistry labs for preparation of ^212^Pb-radiopharmaceuticals use high-purity metal-free starting materials and consumables, and that cross-contamination from shielding is minimised as much as reasonably practicable by ensuring it is suitably coated in plastics or paint or substituted with tungsten.

Despite the level of ^208^Pb present in the experimental conditions in the reported literature, when radiolabelling with this eluate, an A_m_ of ^212^Pb(VM) of 17.8 MBq/nmol was achieved (McNeil et al. 2023), which corresponds to an occupancy of 1.8%. This demonstrates that with this chelator (S-2-(4-Isothiocyanatobenzyl)-1,4,7,10-tetraaza-1,4,7,10-tetra(2-carbamoylmethyl)cyclododecane, referred to as TCMC) under the conditions tested, the level of ^208^Pb present does not sufficiently impede radiolabelling to prevent attainment of the required molar activities for pre-clinical or clinical work. However, with other chelators and under other conditions this may not be the case, and because of the many factors involved, it will be important to collect more experimental data to refine our understanding of the criticality of the ^208^Pb level, which will allow the system to be optimised.

The authors would therefore encourage others to measure and report the level of ^208^Pb (and other metals) in different ^212^Pb generator eluates at different elution times alongside radiolabelling results and the max A_m_ of ^212^Pb(VM) achieved. It would also be useful to assess a range of chelators for ^212^Pb under identical conditions, to see if specific advantages can be gleaned, as has been conducted for ^68^Ga-radiopharmaceuticals (Cusnir et al. 2019; Velikyan et al. 2004).

## Conclusions

Our paper provides a method for tracking the activity of radionuclides on a ^212^Pb parent generator with time, by application of the matrix method described in Ladshaw et al. (2020). The optimal elution time with respect to effective specific activity (t_opt(ESA)_) of ^212^Pb (with respect to the presence of ^208^Pb) was calculated as 16.5 h (61.0% of ^224^Ra starting activity, ESA = 3.32 × 10^16^ Bq/g, A_m_ = 6997 MBq/nmol) for a ^224^Ra generator, and 19.2 h (71.4% of ^228^Th starting activity, ESA of 3.12 × 10^16^ Bq/g, A_m_ = 6566 MBq/nmol for a ^228^Th generator).

Where data is available in the literature (for TCMC chelator) the level of ^208^Pb present is seen not to impede radiolabelling enough to stop the required molar activities for pre-clinical or clinical work being achieved. However, with other chelators and under other conditions this may not be the case. The authors would encourage others to measure and report the level of ^208^Pb (and other metals) in different ^212^Pb generator eluates at different elution times alongside radiolabelling results and the max A_m_ of ^212^Pb(VM) achieved. It would also be useful to assess a range of chelators for ^212^Pb under identical conditions, to see if specific advantages can be gleaned, as has been conducted for ^68^Ga-radiopharmaceuticals (Cusnir et al. 2019; Velikyan et al. 2004).

## Supplementary Information

Additional file 1.

## Data Availability

Not applicable.
