# Supplementary material for: Minimising the impact of stable 208Pb on recovery of 212Pb from a generator
Source: EJNMMI Radiopharm Chem. 2025 Jul 30;10:49. doi: 10.1186/s41181-025-00357-4 (PMC12311066; doi:10.1186/s41181-025-00357-4)
Supplement: Supplementary file 1 — Additional file 1. [file 41181_2025_357_MOESM1_ESM.docx]

**Minimising the impact of stable ^208^Pb on recovery of ^212^Pb from a generator – Supplemental Material 1**

Rachel Roberts^ab^ Tim Carthy^a^, Jennifer Young^b^, Temi Awogboro^a^, Howard Greenwood^a^, Jane Sosabowski^c^, Scott Heath^e^, Francis Livens^b^

^a^ Department of Chemistry, The University of Manchester, Oxford Rd, Manchester, M13 9PL, UK

^b^ United Kingdom National Nuclear Laboratory, Springfields, Salwick, Preston, Lancashire, PR4 0XJ, UK

^c^ Centre for Cancer Biomarkers and Biotherapeutics, Barts Cancer Institute, Queen Mary University of London, London EC1M 6BQ, UK

^d^ School of Biomedical Engineering and Imaging Sciences, King's College London, London, United Kingdom SE1 7EH, UK

^e^ Department of Earth and Environmental Sciences, The University of Manchester, Oxford Rd, Manchester, M13 9PL, UK

Corresponding Author: rachel.roberts-2@student.manchester.ac.uk

**Supplementary Information**

For any given decay chain, the nuclides can be sorted in order of decreasing mass number (ensuring that parent nuclides always have lower indices than their progeny) and indexed accordingly. Table 1 illustrates this for the ^232^U chain as an example.

**Table 1** Nuclides of the ^232^U decay chain using data from [1].

| Index | Nuclide | Half-life (years) | λ (s^-1^) |
| --- | --- | --- | --- |
| 0 | ^232^U | 6.89x10^01^ | 3.19x10^-10^ |
| 1 | ^228^Th | 1.91x10^00^ | 1.15x10^-08^ |
| 2 | ^224^Ra | 9.94x10^-03^ | 2.21x10^-06^ |
| 3 | ^220^Rn | 1.76x10^-06^ | 1.25x10^-02^ |
| 4 | ^216^Po | 4.59x10^-09^ | 4.79x10^00^ |
| 5 | ^212^Pb | 1.21x10^-03^ | 1.81x10^-05^ |
| 6 | ^212^Bi | 1.15x10^-04^ | 1.91x10^-04^ |
| 7 | ^212^Po | 9.44x10^-15^ | 2.33x10^06^ |
| 8 | ^208^Tl | 5.82x10^-06^ | 3.77x10^-03^ |
| 9 | ^208^Pb | 1.00x10^100^ | 2.20x10^-108^ |

Note that although the referenced paper [2] describes a separate matrix solution for stable nuclides, for mathematical simplicity in this work, any stable nuclides in a chain (e.g. ^208^Pb) are given arbitrarily long (but unique within the chain) half-lives. This avoids singularities in the population functions (Eq. 7 & 8) and generates decay constants for the stable nuclides that are so small as to be insignificant in terms of subsequent calculations on applicable timescales.

Once so arranged and given a vector for the starting conditions, the atom count, z, for any nuclide i at any time t can be obtained using Eq 1 taken from p8. of [2]:

$$z_{i}^{t}=\sum_{j\leq i} \left( \sum_{k=j}^{i} v_{i}^{k}u_{k}^{i}e^{{-\lambda}_{k}t} \right)z_{j}^{0}$$

**Eq 1** Atom count z

Where:

*t*=time in seconds,

*i, j* and *k* are nuclide indices,

*λ_k_*=decay constant for nuclide *k*,

*v* & *u* are lower triangular matrices whose values are generated using population functions described later,

And *z*^0^ is the vector of initial atom counts at *t*=0.

The matrix populating function for *v* is given as the following recursive function [2] Eq 2:

$$v_{i}^{k}=\left\{ \begin{aligned} 0 for i<k \\ \\ 1 for i=k \\ \\ -\frac{1}{\lambda_{k}-\lambda_{i}}\sum_{j<i} v_{j}^{k}b_{\mathrm{ij}}\lambda_{j} for i>k \end{aligned} \right.$$

**Eq 2** Population Function for Matrix v

Where *b* is the branching matrix described below.

The matrix populating function for *u* is given as the following recursive function [2] Eq 3:

$$u_{i}^{k}=\left\{ \begin{aligned} 0 for i<k \\ \\ 1 for i=k \\ \\ -\sum_{j<i} u_{j}^{k}v_{i}^{j} for i>k \end{aligned} \right.$$

**Eq 3** Population function for Matrix u

The branching matrix *b* for the ^232^U chain is shown in Table 2:

**Table 2** Branching matrix for the ^232^U chain


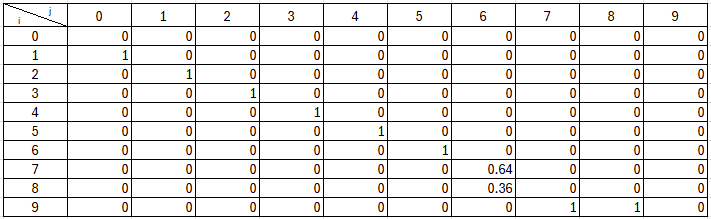


This describes the proportion of the j^th^ nuclide (columns) that decays to the i^th^ nuclide (rows).

For example, the branch at ^212^Bi (j=6) can be seen at b_7,6_ & b_8,6_ and the fact that both these daughter nuclides (j=7 & j=8) decay 100% to ^208^Pb (j=9) is shown at b_9,7_ and b_9,8_.

MathCAD Prime 8 was used to implement the populating functions for *v* & *u* and the resulting matrices for the ^232^U chain are shown below in Table 3 and 4:

**Table 3** Matrix v


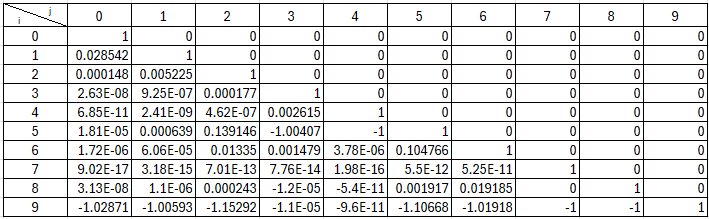


**Table 4** Matrix u


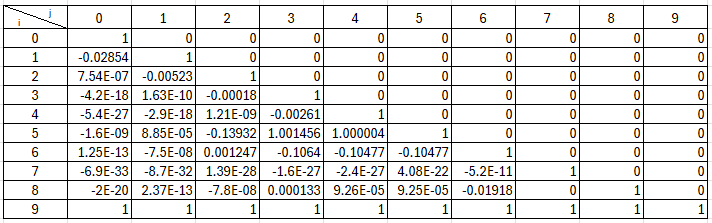


Eq 1 was implemented in Mathcad Prime 8. For any given decay chain the only values required for calculation of the atom count of any nuclide *j* at any time *t* are:

1. the decay constants *λ.*
2. the matrices *v* & *u*.
3. the initial atom count vector *z*^0^.

1 & 2 are constants for a given decay chain and 3 is a simple vector variable.

It is a relatively simple matter to export the constant values generated in Mathcad Prime for a given decay chain to an excel workbook, define a suitable variable for *z*^0^ and implement Eq 1 in Visual Basic for Applications (VBA). This was implemented and the results obtained again agreed closely with these calculated by other means.

**References**

1. Tuli JK. Nuclear Wallet Cards. National Nuclear Data Center. 2011. [https://www.nndc.bnl.gov/walletcards/doc/wallet-cards-2011-11.pdf. Accessed 13 Feb 2025](https://www.nndc.bnl.gov/walletcards/doc/wallet-cards-2011-11.pdf.%20Accessed%2013%20Feb%202025).

2. Ladshaw A, Wiechert AI, Kim YH, Tsouris C, Yiacoumi S. Algorithms and algebraic solutions of decay chain differential equations for stable and unstable nuclide fractionation. Comput. Phys. Commun. 2020; https://doi.org/10.1016/j.cpc.2019.106907
